# Binding of IgA1 and surface-expressed collagen-binding protein of *Streptococcus mutans* contributes to IgA nephropathy pathogenesis

**DOI:** 10.3389/fcimb.2025.1673581

**Published:** 2026-01-07

**Authors:** Daiki Matsuoka, Kana Suehara, Shuhei Naka, Taro Misaki, Yasuyuki Nagasawa, Seigo Ito, Yuto Suehiro, Ryota Nomura, Kazuhiko Nakano, Michiyo Matsumoto-Nakano

**Affiliations:** 1 Department of Pediatric Dentistry, Okayama University Graduate School of Medicine, Dentistry and Pharmaceutical Sciences, Okayama, Okayama, Japan; 2 Division of Nephrology, Seirei Hamamatsu General Hospital, Hamamatsu, Shizuoka, Japan; 3 Department of Nursing, Faculty of Nursing, Seirei Christopher University, Hamamatsu, Shizuoka, Japan; 4 Department of General Internal Medicine, Hyogo Medical University, Nishinomiya, Hyogo, Japan; 5 Department of Internal Medicine, Japan Self-Defense Force Iruma Hospital, Saitama, Japan; 6 Department of Pediatric Dentistry, Graduate School of Dentistry, The University of Osaka, Suita, Osaka, Japan; 7 Department of Pediatric Dentistry, Graduate School of Biomedical and Health Sciences, Hiroshima University, Hiroshima, Japan

**Keywords:** bacterial surface proteins, collagen-binding protein, human immunoglobulins, IgA nephropathy, *Streptococcus mutans*

## Abstract

**Background:**

The present study was conducted to examine the interaction between collagen-binding protein (Cnm) of *Streptococcus mutans* and immunoglobulin (IgA) to clarify the possible involvement in IgA nephropathy (IgAN) development.

**Methods:**

The binding of Cnm to human immunoglobulins was examined using an enzyme-linked immunosorbent assay. A nephritis-induced rat model was employed to confirm the localization of Cnm.

**Results:**

IgA1 showed significantly greater binding ability to Cnm than to other bacterial surface proteins, and Cnm showed significantly greater binding ability to IgA1 than to other immunoglobulins. In rats administered Cnm, IgA deposition was observed in the glomerular mesangial region. Furthermore, biotin-labeled Cnm was observed in the same region as IgA deposition in the Cnm group.

**Conclusions:**

Taken together, it is considered that following invasion into the bloodstream, Cnm binds to and forms a complex with IgA1, leading to deposition of IgA1 in renal glomeruli.

## Introduction

IgA nephropathy (IgAN) is the most common form of chronic glomerulonephritis, in which immunoglobulins, mainly IgA, are deposited specifically in the mesangial region of the glomerulus, and mesangial cell proliferation and increased mesangial matrix expansion are observed (Berger, 1969; Wyatt and Julian, 2013; Yeo et al., 2018). The pathogenesis of IgAN has not been clarified, although it is considered that mucosal immunity may be involved because there are cases of genetic predisposition, as well as aggravation during upper airway infections (Coppo et al., 2010; Suzuki et al., 2011b; Donadio and Grande, 2002). Although association with various bacterial and viral infections have been reported, such as Cytomegalovirus (Gregory et al., 1988; Waldo et al., 1989), Epstein–Barr virus (Iwama et al., 1998), *Haemophilus parainfluenzae* (Suzuki et al., 1994), *Staphylococcus aureus* (Koyama et al., 2004), and streptococcal M protein (Schmitt et al., 2010), it is unclear whether these bacterial and virus form immune complexes with IgA1 and are deposited in glomeruli, whether abnormalities in mucosal immunity result in the production of glomerularly deposited IgA1, or whether the deposition is specific.

*Streptococcus mutans*, a major cause of dental caries, is a gram-positive, facultative anaerobic bacterium (Hamada and Slade, 1980) that can induce infective endocarditis by entering the bloodstream during invasive dental procedures, such as tooth extraction (Nakano and Ooshima, 2009). The collagen-binding protein, Cnm, which is approximately 120 kDa in size, is expressed on the surface of 10%–20% of *S. mutans* strains (Sato et al., 2004), and plays a role in adhesion and invasion of vascular endothelial cells, suggesting that it may be an important factor in infective endocarditis (Abranches et al., 2011; Nomura et al., 2013, 2020). The fundamental structure of Cnm consists of a collagen-binding domain (domain A) at its N-terminal region and a repeating amino acid sequence (B-repeat) at its C-terminal region. Furthermore, the presence of an LPXTG sequence at the C-terminus indicates that this is a cell-wall-anchored protein. The LPXTG motif is covalently linked to the peptidoglycan layer in the cell wall (Sato et al., 2004). The PgfS enzyme mediates glycan modification of Cnm, which enhances protein stability and enables functions related to pathogenicity, such as collagen binding and cell invasion (Di Cologna et al., 2021). Such modification is considered to be a key factor for adhesion of bacteria and their invasion into host tissues. It has been reported that *S. mutans* expressing Cnm is detected more frequently in IgAN patients than in healthy controls (Misaki et al., 2015). In fact, intravenous administration of *S. mutans* expressing Cnm isolated from the oral cavity of IgAN patients induced IgAN-like nephritis in rats (Naka et al., 2020). In addition, when Cnm-positive *S. mutans* was inoculated into the oral cavity of a rat caries model, the organism caused severe caries extending into the pulp cavity and IgAN-like lesions (Naka et al., 2021). More recently, animal studies using Cnm-deficient mutant strains and recombinant Cnm (rCnm) protein (Naka et al., 2022) have demonstrated that Cnm itself is present in glomeruli and causes IgAN-like nephritis (Naka et al., 2024).

The Cnm protein has been found to be localized on the surface of bacterial cells in a form that can bind to other bacteria (Naka et al., 2022), and is thereby thought to be involved in the development of systemic diseases by binding to collagen, blood vessels, and cells through its collagen-binding ability (Nakano et al., 2011; Naka et al., 2014, 2016, 2018). Furthermore, the structure of Cnm, in its bacterial binding form, has a prominent protruding shape that confers its ability to bind to molecules (Naka et al., 2022). These findings suggest that Cnm may be involved in the pathogenesis of IgAN by forming immune complexes with IgA, which are then deposited in glomeruli. In this study, we focused on the binding properties of Cnm to IgA1 and report a possible mechanism for the development of IgAN.

## Materials and methods

### Bacterial strains and recombinant proteins

The Cnm*-*positive *S. mutans* strain SN74 (serotype *e*) was isolated from the oral cavity of a patient with severe IgAN (Naka et al., 2020). Other Cnm-positive *S. mutans* strains used were TW295, TW871 (Fujiwara et al., 2001), LJ24, LJ32 (Nakano et al., 2007), NN2007, and NN1141 (Nakano et al., 2004). The Cnm-negative *S. mutans* strain MT8148 (serotype *c*) isolated from a Japanese child was used as a reference strain (Ooshima et al., 1983). These strains were cultured on Mitis–Salivarius agar (Difco Laboratories, Detroit, MI, USA) plates containing bacitracin (0.2 U/mL; Sigma Chemical Co., St. Louis, MO, USA) or in brain heart infusion (BHI; Difco) broth or Todd–Hewitt (TH; Becton Dickinson) broth. Strain SN74CND, a Cnm-inactivated isogenic mutant strain of SN74, and SN74CND comp, a Cnm complemented strain of SN74CND, were constructed in our previous study (Naka et al., 2022). We used the recombinant Cnm protein (rCnm) extracted after the transformation of *Escherichia coli* BL21 (DE3) (Nippon Gene, Tokyo, Japan) with a plasmid containing the *cnm* gene inserted into a pGEX 6P-1 (Cytiva, Tokyo, Japan) vector (Naka et al., 2022). In addition, rGTFB (Matsumi et al., 2015), a recombinant protein extracted after transformation of *E. coli* BL21 strain in which a plasmid containing the *gtfb* gene was inserted into the pGEX 6P-1 vector, and rGbpC (Takashima et al., 2015), a recombinant protein extracted after transformation of *E. coli* BL21 strain in which a plasmid containing the *gbpC* gene was inserted into the pET42a vector were used. Expression of these recombinant proteins was confirmed prior to their use in this study (**Supplementary Figure 1**).

### Expression of the *cnm* gene and Cnm under different environmental conditions

*S. mutans* SN74 overnight cultures were diluted 1:100 in sterile BHI broth and grown to mid-exponential growth phase (OD600 of 0.6) under different environmental conditions (anaerobic, aerobic, and various pH values). Total RNA was isolated from 15 mL of log-phase cell cultures. For reverse transcription-PCR (RT-PCR) (Nomura et al., 2005) analysis, RNA samples were treated for 15 min at 37°C with 1.0 U of RNase-free DNase (Amersham Biosciences Corp., Piscataway, NJ, USA) per mL to remove contaminating DNA. Reverse transcription was carried out with Super Script III (Invitrogen) according to the instructions of the supplier. Real-time RT-PCR was performed using cDNA samples with either 16S rRNA (**Table 1**) or *cnm* specific primers (**Table 1**) using IQ-Supermix PCR reagent (Bio-Rad) in an iCycler thermal cycler according to the manufacturer’s recommendations. Relative expression levels of the *cnm* gene transcripts were then calculated by normalizing the levels of the specific RNA of the *cnm* gene with the level of 16S rRNA. After normalizing Ct values for *cnm* genes to the total amount of 16S rRNA, all samples were compared and relative fold changes in the samples were calculated using the -ΔΔCt method with an MyIQ real-time PCR detection system (BioRad Laboratories). Western blot analysis of cells of *S. mutans* strain SN74 cultured in different pH environments was performed using rabbit anti-Cnm antibodies (Naka et al., 2022). Next, bands were visualized using the alkaline phosphatase-conjugated anti-rabbit immunoglobulin G antibody (New England Biolabs, Beverly, MA, USA) and 5-bromo-4-chrolo-3-indolylphosphate-nitro-blue tetrazolium substrate (Bio-Rad). After visualizing, EDTA (0.5 M, pH8.0) was added to all wells.

**Table 1:** Primers used for qRT-PCR in this study.

Name	Sequence (5’→3’)	Reference
16SrRNA-F	CAG CGC AGC TGA TAG CTG TTT GT CT	Matsumoto-Nakano and Kuramitsu, 2006
16SrRNA-R	CTG CTG GCA AAT TCG CTT ACT TG	
cnm-CF	CTA CCG TTT TCT ACT ATA AGA CTG GGG	Naka et al., 2016
cnm-CR	CCT TCT TGA CCG CGA TAA GAC TCA CTG CCA	

### Cnm expression at different incubation times

*S. mutans* SN74 were grown in BHI broth at 37°C for 12, 24, or 48 h. The cells were collected by centrifugation and washed with phosphate-buffered saline (PBS; pH 7.4), then re-suspended with PBS for a final dilution in 1x SDS gel-loading buffer, while the supernatants were concentrated by 30% ammonium sulfate precipitation and dissolved in the same buffer. An equal amount of each protein was separated by 7.5% SDS-polyacrylamide gel electrophoresis and then transferred onto polyvinylidene difluoride membranes (Immobilon; Millipore, Bedford, MI, USA). Transferred protein bands were exposed to anti-rabbit antibodies against Cnm (Naka et al., 2022). Next, bands were visualized using the alkaline phosphatase-conjugated anti-rabbit immunoglobulin G antibody (New England Biolabs, Beverly, MA, USA) and incubate the membrane with the substrate (Pierce™ ECL Plus Western Blotting Substrate) (Thermo Fisher Scientific, Waltham, MA) working solution for 5 minutes. After visualizing, image the blot using an imaging system (FUSION SOLO. 6S. EDGE, M&S Instruments Inc., Tokyo, Japan).

### Scanning electron microscopy

SEM was performed according to the protocol described by Yuan et al. (2021) (Yuan et al., 2021) to observe morphologic changes in the tested strains. Overnight cultures of *S. mutans* were harvested and pre-fixed with 2% glutaraldehyde and 2% paraformaldehyde at 4°C for 16 h, then washed with 0.1 M phosphate buffer (pH 7.4), fixed with 2% osmium tetroxide for 1.5 h, and washed with 0.1 M phosphate buffer (pH7.4). Then, samples were dehydrated using an ethanol gradient, immersed in t-butyl alcohol for 30 min, and dried with CO_2_ for 2 h. The prepared specimens were placed on aluminum stubs, coated with osmium (HPC-IS, Vacuum Device, Ibaraki, Japan) and observed by SEM (S-4500, Hitachi, Tokyo, Japan).

### Enumeration of bacterial numbers at different incubation times

To evaluate the viability of bacteria during incubation, pre-grown strain SN74 were inoculated into BHI medium and incubated for 12, 24 or 48 h. Then, 100 μL of culture was serially diluted and inoculated onto Mitis–salivarius agar plates. The plates were incubated at 37°C for 48 h and then colonies were counted.

### Binding assay of bacterial surface proteins to human immunoglobulins

Human IgA1 (Native Human IgA1 protein: ab91020), IgA2 (Native Human IgA2 protein: ab91021), or IgG (Native Human IgG protein: ab91102) protein and rCnm, rGTFB, or rGbpC were suspended in PBS to 0.1 μg/μL. Human IgA1, IgA2 and IgG proteins were used to coat ELISA plates (100 μl per well) and plates were incubated at 4°C for 16 h. The wells were washed three times with distilled water (DW), then blocking buffer (5.0% skim milk) was added and the plates were incubated at 37°C for 2 h. After washing again (DW), rCnm, rGTFB, or rGbpC was added (100 μL per well) and incubated for 3 h at 25°C with shaking. After another wash with DW, anti-rabbit antibodies against Cnm, GTFB, and GbpC, and the alkaline phosphatase-conjugated anti-rabbit immunoglobulin G antibody were added to all wells and incubated for 1 h at 25°C. After a final wash with DW, a color detection solution (5-bromo-4-chrolo-3-indolylphosphate-nitro-blue tetrazolium substrate) was applied, as recommended by the supplier, and the samples were incubated. After visualizing the plates, EDTA (0.5 M, pH8.0) was added to all wells. The subsequent OD570 results were determined using a microplate reader. All assays were carried out three times.

### Slot blot assay of IgA1 binding to rCnm

The binding ability of human IgA1 protein to rCnm was examined using slot blot assays (Matsumoto-Nakano et al., 2008). Briefly, rCnm was blotted onto polyvinylidene difluoride membranes and reacted with human IgA1 protein for 10 min at room temperature. The membrane was washed with PBS, then incubated with blocking buffer (5.0% skim milk) for 1 h at 25°C. After another wash with DW, anti-rabbit antibodies against Cnm and the alkaline phosphatase-conjugated anti-goat immunoglobulin G antibody were added to all wells and incubated for 1 h at 25°C. The intensity of the signal on membranes was determined using ImageJ software (Version 1.43, Macintosh computer application, Scion, MD, USA). After visualizing the membrane, EDTA (0.5 M, pH 8.0) was added.

### Binding assay of the IgA1 in the human plasma of IgAN patients to rCnm

This study protocol fully adhered to the Declaration of Helsinki (64th WMA General Assembly, Fortaleza, Brazil, 2013) and was approved by the Ethics Committee of Okayama University Graduate School of Medicine, Dentistry and Pharmaceutical Sciences (approval no. 1704-036), Seirei Hamamatsu General Hospital (approval no. 2315).

First, rCnm was suspended in PBS to 0.1 μg/μL, and was then used to coat ELISA plates (100 μL per well) and incubated at 4°C for 16 h. The wells were washed three times with DW, then blocking buffer (5.0% skim milk) was added and the plates were incubated at 37°C for 2 h. After wash again (DW), the plasma of IgAN patients was added (200 μL per well) and incubated for 3 h at 25°C with shaking. After another wash with DW, anti-human IgA and the alkaline phosphatase-conjugated anti-goat immunoglobulin G antibody were added to all wells and incubated for 1 h at 25°C. After a final wash with DW, a color detection solution (3,3′, 5,5′-tetramethylbenzidine) was applied for incubation, as recommended by the supplier. After visualizing the plates, 1 M HCl was added to all wells. The subsequent OD430 results were determined using a microplate reader. All assays were carried out three times.

### Visualization of the binding of *S. mutans* to IgA1 by fluorescent dye labeling

Each test organism was grown overnight in TH broth and the turbidity was determined and a solution of OD550 nm = 1.0 was prepared with PBS. After fluorescent staining of the bacteria with SYTO9^®^, each test sample was fixed to a cover glass, and then IgA1 labeled with an Alexa Fluor^®^ Antibody Labeling Kits was added and allowed to react for 3 h. After washing, imaging was performed using an LSM510 confocal laser scanning microscope (version 4.2, Carl Zeiss MicroImaging Co., Ltd., Germany) at a laser wavelength of 543 nm.

### Preparation of biotin-labeled rCnm protein

A sample solution containing wash buffer (100 μL) and rCnm protein (200 μg) was added to a filtration tube, mixed by pipetting, then centrifuged at 8,000 × g for 10 min. Next, 10 µL of DMSO was added to NH_2_-reactive biotin and dissolved by pipetting. Reaction buffer (100 μL) was added to the filtration tube, then 8 µL of NH_2_-reactive biotin solution was added, mixed with a pipette, and incubated at 37°C for 10 min. Next, 100 μL of wash buffer was added to the filtration tube and centrifuged at 8,000 × g for 10 min. The filtrate was discarded and 200 μL of wash buffer was added to the filtration tube and centrifuged at 8,000 × g for 10 min. This step was repeated. Then, 200 μL of wash buffer was added, and after 10 rounds of pipetting the conjugate was recovered and transferred to a microtube, followed by storage at 4°C.

### Evaluation using an IgAN-like lesion-induced rat model

In this experiment, all rats were treated humanely, in accordance with the National Institutes of Health and AERI-BBRI Animal Care and Use Committee guidelines. All procedures were approved by the Animal Care and Use Committee of Okayama University (approval number: OKU2020864). The effects of intravenous administration of *S. mutans* were analyzed in a rat model, as described previously, with some modifications (Naka et al., 2020). Briefly, specific-pathogen-free Sprague–Dawley rats (male, 4 weeks old; Japan CLEA, Tokyo, Japan) were randomly divided into PBS (n=5) and biotin-labeled rCnm (n=10) groups. Rats were allowed free access to water and food throughout the experimental period. They were fed an MF diet (ORIENTAL YEAST Co., Ltd, Tokyo, Japan). Rats received intravenous injections (through the jugular vein) of PBS alone or 200 μg of biotin-labeled rCnm protein. At 45 days post-infection, the animals were euthanized and their kidneys were removed. Euthanasia was performed according to the AVMA Guidelines for the Euthanasia of Animals 2020. These animals were rendered unconscious and their cardiac or pulmonary functions were stopped using carbon monoxide (CO) and carbon dioxide gas (CO_2_), with methods that caused the less possible pain.

Histological evaluations of the kidneys were performed using the following methods. Excised kidney samples were fixed in 3.7% formaldehyde (diluted in PBS), embedded in paraffin, and cut into 3 μm-thick sections for histopathological analysis. PAS staining was performed to evaluate increases in the numbers of mesangial cells and the mesangial matrix in glomeruli (data not shown). Additionally, alterations in IgA and Cnm expression patterns in tissue samples were detected using standard immunohistochemical techniques with IgA and Cnm-specific antibodies. The primary antibodies used were purified mouse anti-rat IgA (BD Biosciences, Franklin Lakes, NJ, USA), and anti-rCnm antibodies. Secondary antibodies were donkey anti-mouse IgG H&L (Alexa Fluor 488) preadsorbed (ab150109; Abcam) and donkey anti-rabbit IgG H&L (Alexa Fluor 647) (ab150075; Abcam). Fluorescence immunostaining was performed using these antibodies. Stained sections were observed using a semi-motorized fluorescence microscope (BX53; OLYMPUS, Tokyo, Japan).

### Statistical analyses

Statistical analyses were performed using GraphPad Prism 8 Statistics Software (GraphPad, Inc., La Jolla, CA, USA). All results are presented as the mean ± standard deviation (SD). The *cnm* gene expression levels of strain SN74 strain and other Cnm-positive strains in anaerobic and aerobic culture, as determined by real-time quantitative RT-PCR were compared using the Student’s *t-*test. *P-*values less than 0.05 were considered statistically significant. The abilities of IgA1 to bind to rCnm in the plasma of IgAN patients and healthy subjects were compared using the Student’s *t-*test. *P-*values less than 0.05 were considered statistically significant. The *cnm* gene expression levels of SN74 strains under different pH environments using real-time quantitative RT-PCR were compared by analysis of variance (ANOVA) with Bonferroni’s correction. *P-*values less than 0.006 were considered statistically significant. The expression levels of Cnm of strain SN74 in different pH environments were compared by western blot analysis and differences were assessed by ANOVA with Bonferroni’s correction. *P-*values less than 0.006 were considered statistically significant. The binding of IgA1 to bacterial surface proteins of *S. mutans* was assessed by ANOVA with Bonferroni’s correction. *P-*values less than 0.013 were considered statistically significant. The binding of rCnm to immunoglobulins was assessed by analysis of ANOVA with Bonferroni’s correction. *P-*values less than 0.008 were considered statistically significant.

## Results

### Expression of the *cnm* gene of strain SN74 under different culture conditions

**Figure 1:**
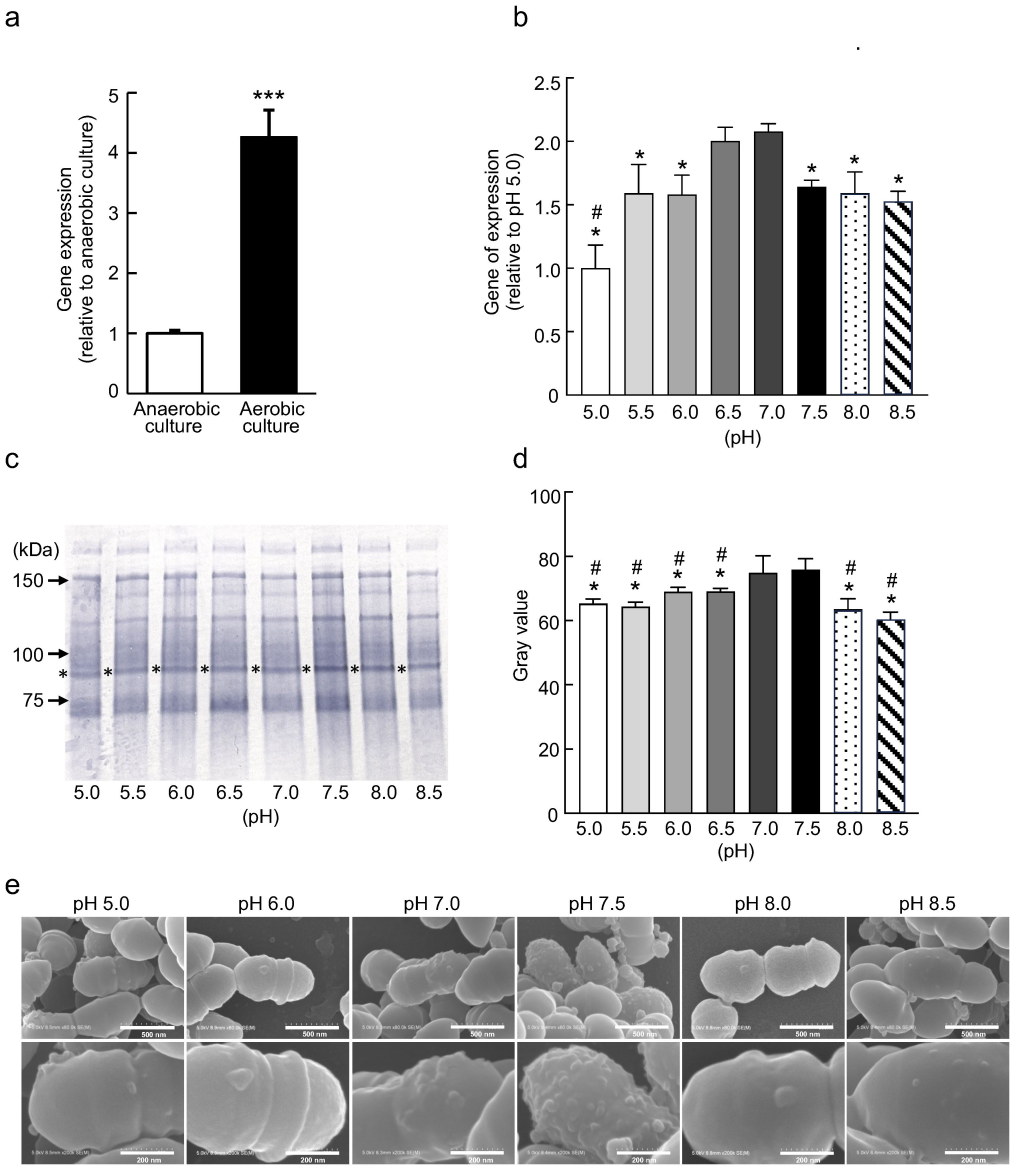
Analysis of Cnm expression under different environmental conditions. **(a)** Comparison of the *cnm* gene expression levels of strain SN74 strain in anaerobic and aerobic cultures by real-time quantitative reverse transcription PCR. The mRNA expression values were quantified by the -ΔΔCT method using 16S rRNA as an internal control. Fold changes are relative to anaerobic culture. Data are presented as the mean ± SD from five independent experiments. Statistical significance was determined using the Student’s *t*-test. *P < 0.05. **(b)** Comparison of the *cnm* gene expression levels of strain SN74 in culture media of different pH by real-time quantitative reverse transcription PCR. The mRNA expression values were quantified by the -ΔΔCT method using 16S rRNA as an internal control. Fold changes are relative to pH 5.0. There were significant differences in the values between pH 7.0 and other pH conditions (*P < 0.006), pH 7.5 and other pH conditions (*P < 0.006). Data are presented as the mean ± SD from three independent experiments. Statistical significance was determined by analysis of variance with Bonferroni’s correction. **(c)** Western blot analysis of the expression of Cnm in strain SN74 under different pH conditions. The band corresponding to the Cnm protein (~90 kDa) is indicated with an asterisk. **(d)** Densitometric analysis of Cnm expression by western blot analysis. There were significant differences in the values between pH 7.0 and other pH conditions (*P < 0.006), and pH 7.5 and other pH conditions (^#^*P* < 0.006). Data are presented as the mean ± SD from three independent experiments. Statistical significance was determined by analysis of variance with Bonferroni’s correction. **(e)** Scanning electron microscopic images of strain SN74. Scale bars are 500 nm (upper panels) and 200 nm (lower panels). Magnification of the upper images, 80,000×; magnification of the lower images, 200,000×.

Quantitative PCR (qPCR) analysis revealed that the expression levels of the *cnm* gene in strain SN74 were significantly higher (4.23-fold) under aerobic conditions than under anaerobic conditions (*P* < 0.001) (**Figure 1A**). Furthermore, the *cnm* gene expression levels of the other Cnm-positive *S. mutans* strains (TW295, TW871, LJ24, LJ32, NN2007, NN1141) were all significantly higher under aerobic conditions than under anaerobic conditions (*P* < 0.01) (**Supplementary Figure 2**). When comparing eight different pH incubation conditions, the highest *cnm* expression levels were observed at pH 7.0, with significantly lower expression levels at pH 5.0, 5.5, 6.0, 7.5, 8.0, and 8.5. (*P* < 0.006) (**Figure 1B**). In addition, *cnm* gene expression was significantly higher when cultured at pH 7.5 compared with pH 5.0 (*P* < 0.006) (**Figure 1B**).

Western blot analysis of cells of *S. mutans* strain SN74 cultured in different pH environments was performed using rabbit anti-Cnm antibodies. A positive band was detected at approximately 90 kDa, and the band was more intense for cells cultured at pH 7.0 and 7.5 compared with pH 5.0, 5.5, 6.0, 6.5, 8.0, and 8.5 (**Figure 1C**). Quantification of the bands using ImageJ software showed that the expression levels of Cnm were significantly higher in the pH 7.0 environments than in the pH 5.0, 5.5, 6.0, 6.5, and 8.5 environments (*P* < 0.006). In addition, the expression levels of Cnm were significantly higher in the pH 7.5 environments than in the other pH environments (*P* < 0.006) (**Figure 1D**).

In scanning electron microscopy (SEM) images of *S. mutans* cultured under different pH environments, bumpy structures were observed on the surface of all cells. However, these structures were more numerous and varied in size on the cell surface of *S. mutans* cultured at pH 7.0 and 7.5 compared with those cultured under other pH conditions (**Figure 1E**).

### Bacteria cell numbers during incubation

The number of *S. mutans* strain SN74 cells bacteria during the 48-h incubation period was monitored. We found that cell numbers steadily decreased from 5.5 × 10^8^ CFU at 12 h, to 5.0 × 10^7^ CFU at 24 h, and 3.5 × 10^6^ CFU at 48 h (**Supplementary Figure 3**).

### Cnm expression in the bacterial cells and supernatants

**Figure 2:**
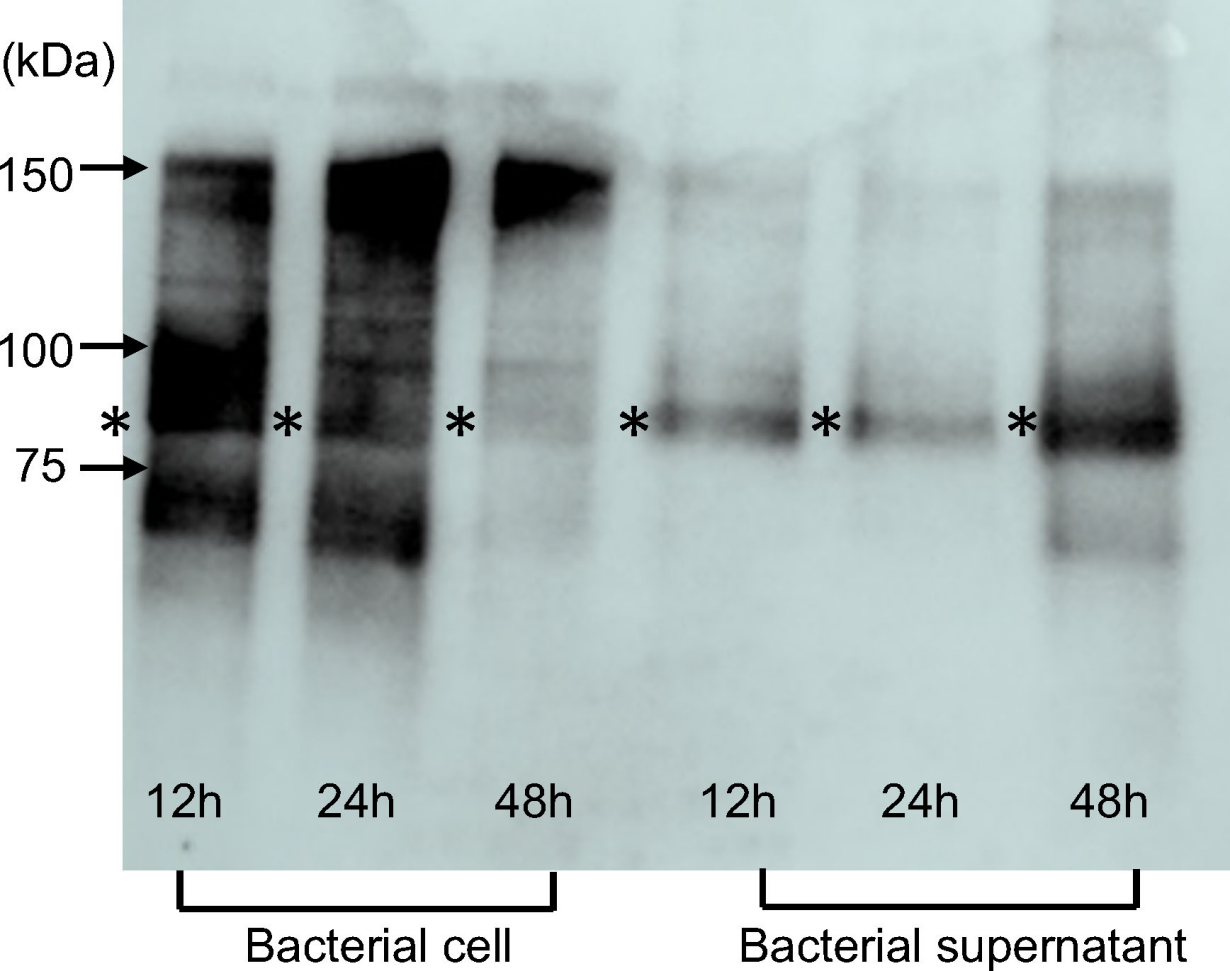
Comparison of Cnm expression in bacteria cells and bacterial supernatants by chemiluminescence-based western blot analysis. The band corresponding to the Cnm protein (~90 kDa) is indicated with an asterisk.

Western blot analysis of bacterial cells using rabbit anti-rCnm antibody revealed an intense band at a molecular weight of approximately 90 k-Da after 12 and 24 h of incubation, and this band appeared weaker after 48 h (**Figure 2**). By contrast, when analyzing the bacterial cell supernatant, a weak band at ~90 k-Da was observed after 12 and 24 h of incubation, and this band appeared more intense after 48 h.

### Binding experiments

**Figure 3:**
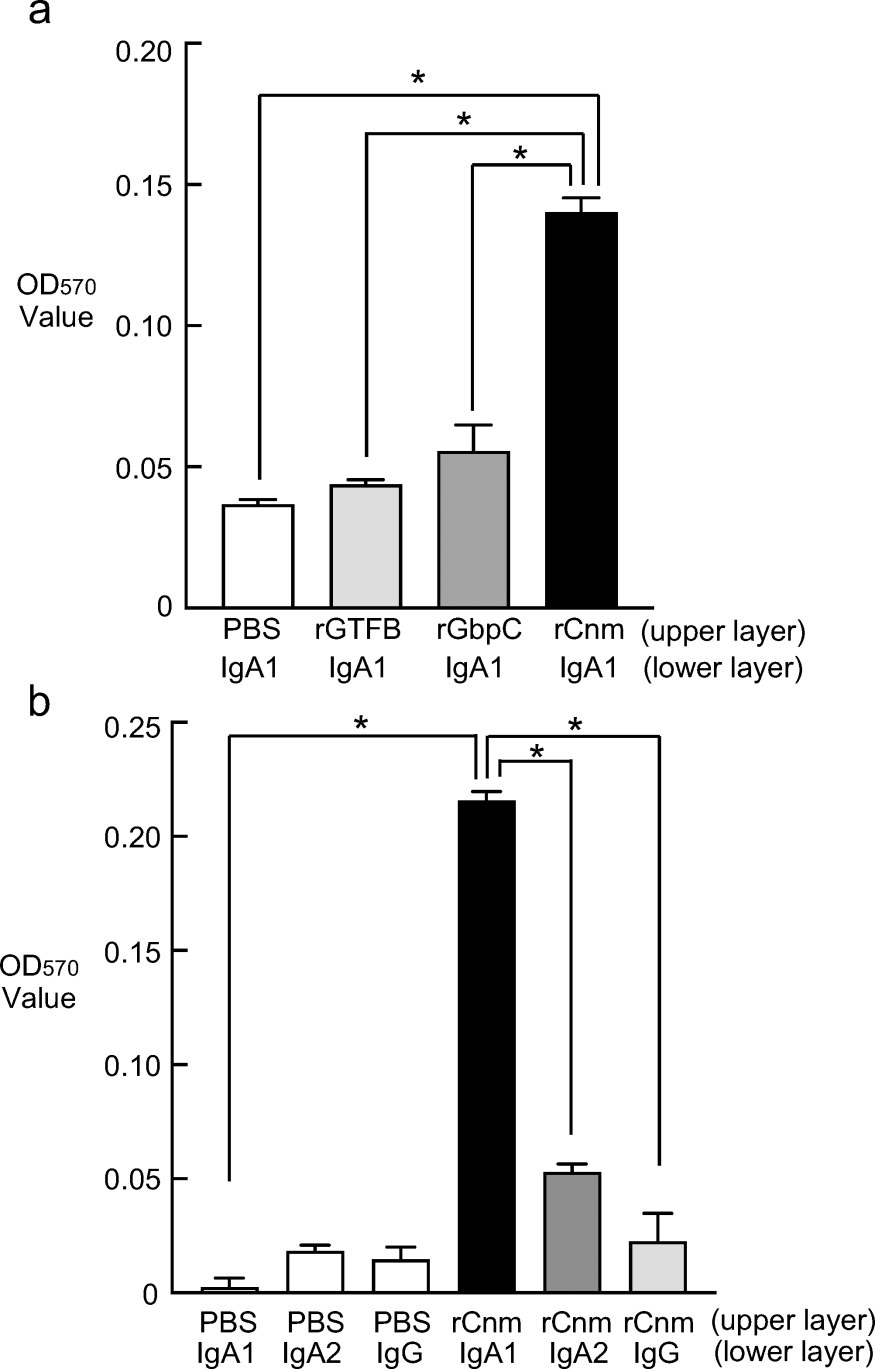
Analysis of human immunoglobulin binding to *S. mutans* bacterial surface proteins. **(a)** Binding experiments between *S. mutans* surface proteins (rGTFB, rGbpC, rCnm) and IgA1. Data are presented as the mean ± SD from four independent experiments. Statistical significance was determined by by analysis of variance with Bonferroni’s correction. *P < 0.013. The lower layer of the ELISA plate was coated with IgA1 and the upper layer was conjugated with PBS, rGTFB, rGbpC, and rCnm protein. **(b)** Binding experiments of Cnm to immunoglobulins (IgA1, IgA2, IgG). The lower layer of the ELISA plate was coated with IgA1, IgA2, IgG and the upper layer was conjugated with PBS and rCnm protein. Data are presented as the mean ± SD from four independent experiments. Statistical significance was determined by analysis of variance with Bonferroni’s correction. *P < 0.008.

A comparison of IgA1 binding to the bacterial surface proteins of *S. mutans* revealed significantly higher OD570 values when binding to rCnm (0.140 ± 0.005) compared with PBS (0.037 ± 0.002), rGTFB (0.044 ± 0.002), and rGbpC (0.056 ± 0.005) (*P* < 0.013) (**Figure 3A**). These findings suggested that IgA1 binds specifically to Cnm and not the rGTFB and rGbpC proteins tested.

Next, analysis of the binding of rCnm to human immunoglobulins indicated that the OD570 value was significantly higher with IgA1 (0.216 ± 0.004) than with PBS (0.002 ± 0.004), IgA2 (0.053 ± 0.003), or IgG (0.023 ± 0.004) (*P* < 0.008) (**Figure 3B**). This finding suggested that Cnm bind specifically to IgA1 among the other immunoglobulins tested. The binding ability of IgA to rCnm was next examined using a dot-blot assay. A concentration-dependent decrease in signal intensity on the membrane was observed for IgA1 reacting with rCnm. The binding rate of IgA1 to rCnm tended to increase in a dose-dependent manner (**Supplementary Figure 4**).

**Figure 4:**
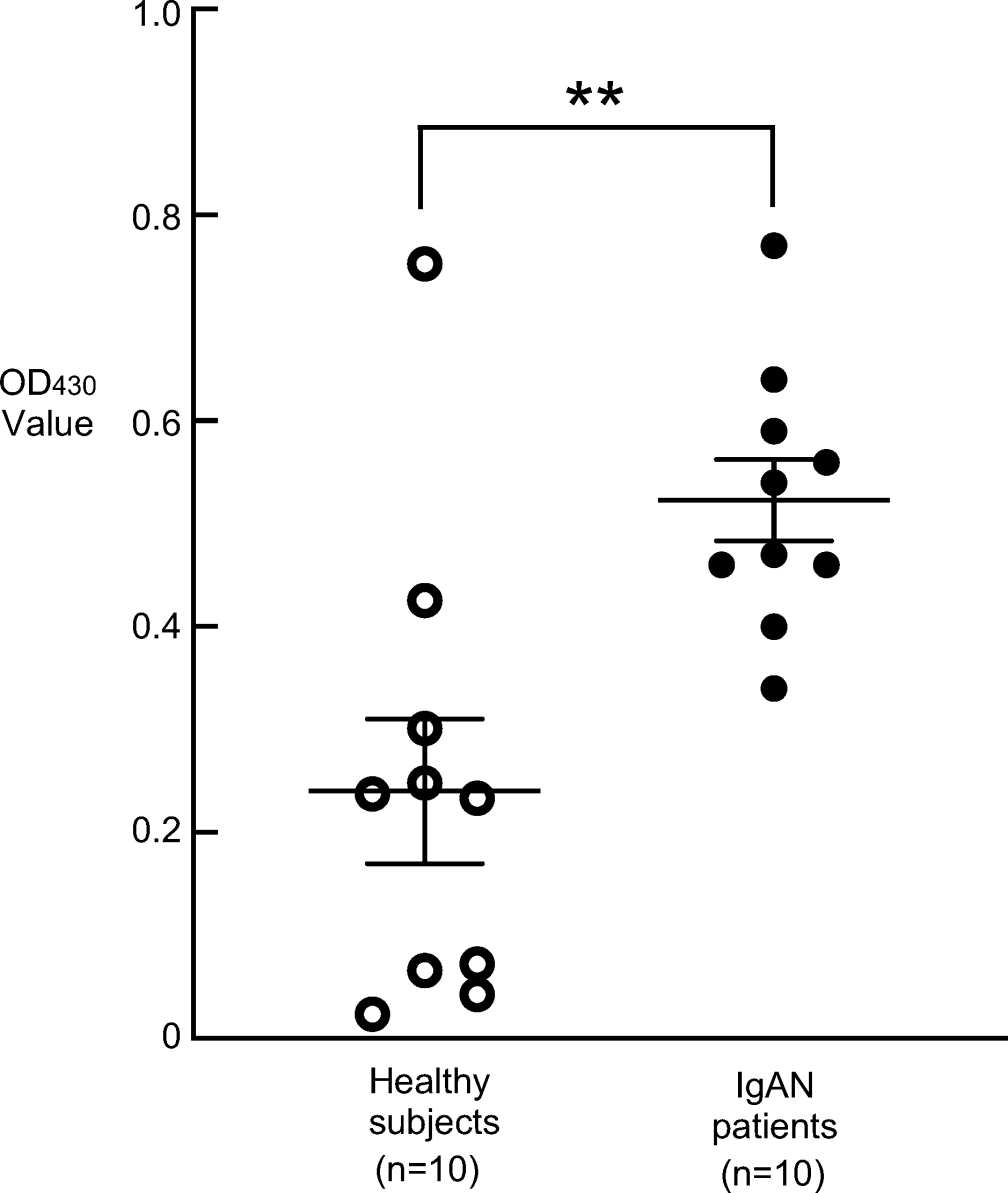
Binding assay of IgA1, from the plasma of IgAN patients and healthy subjects, to rCnm. Data are presented as the mean ± SE from four independent experiments. Statistical significance was determined using the Student’s *t*-test. *P < 0.05.

We next investigated the binding of IgA1 to rCnm in the plasma of patients with IgAN and healthy subjects. As shown in the **Table 2**, the data of the plasma samples from individuals with IgAN or healthy subjects, which were used in this study. The OD430 values of IgA1 and rCnm tended to be higher in plasma of the IgAN patient group compared with the healthy control group. Furthermore, the OD430 values of the IgAN patient group were significantly higher than those of the healthy subjects (*P* < 0.001) (**Figure 4**).

**Table 2:** Age, sex, and serum IgA levels of healthy subjects and IgAN patients enrolled in this study.

Sample no.	Age	Sex	Serum IgA level (mg/dL)
Healthy 1	54	F	−
Healthy 2	36	M	−
Healthy 3	48	M	−
Healthy 4	47	F	−
Healthy 5	42	M	−
Healthy 6	49	F	−
Healthy 7	30	F	−
Healthy 8	28	F	−
Healthy 9	31	M	−
Healthy 10	30	M	−
Patient 1	36	F	189
Patient 2	62	M	377
Patient 3	58	M	318
Patient 4	63	F	730
Patient 5	45	F	365
Patient 6	66	M	395
Patient 7	48	F	283
Patient 8	24	M	284
Patient 9	56	F	211
Patient 10	47	F	289

### Visualization of the binding of *S. mutans* and IgA1 by fluorescent dye labeling

**Figure 5:**
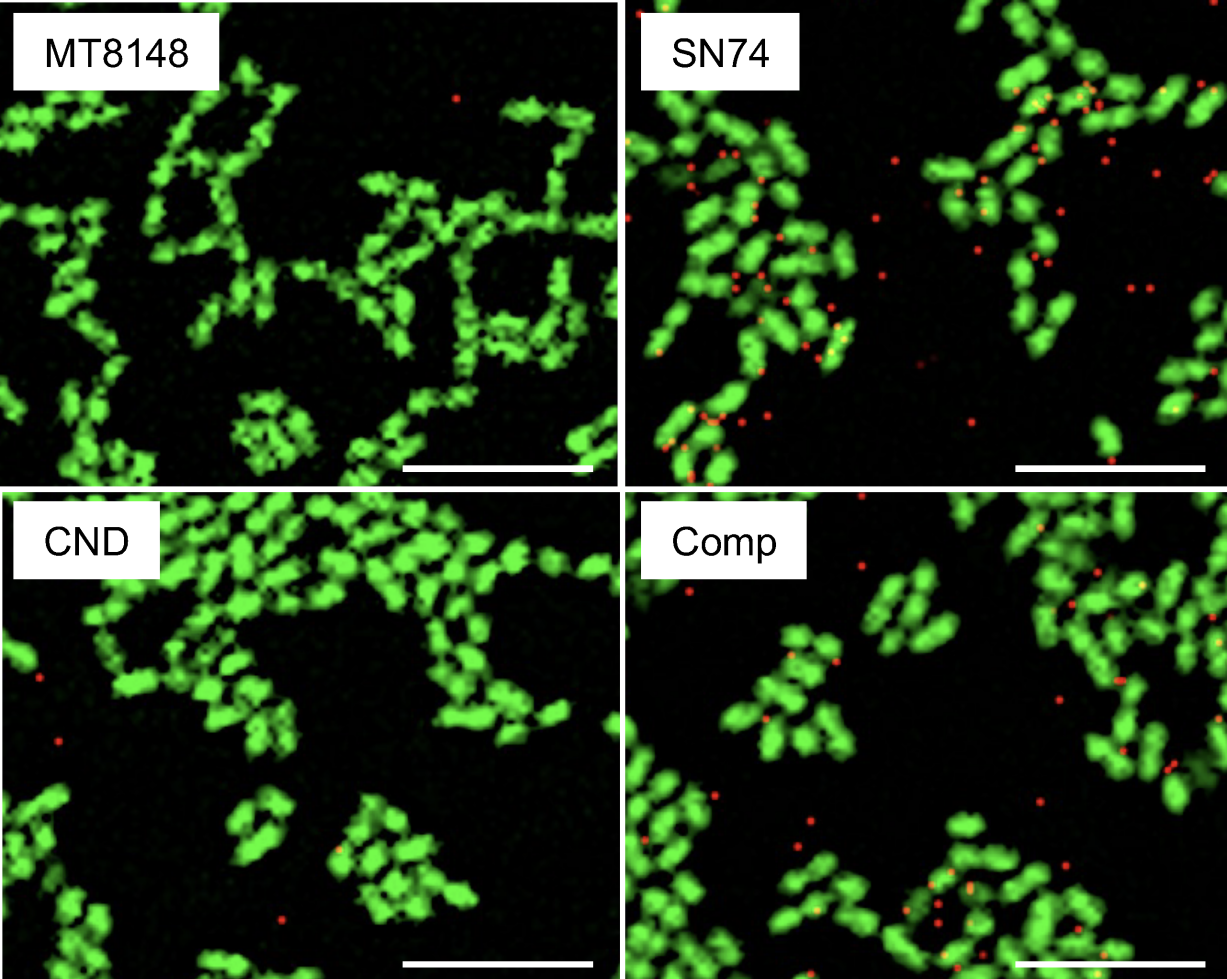
Visualization of the binding of *S. mutans* and IgA1 by fluorescent dye labeling. *S. mutans* is indicated in green and IgA1 is indicated in red. All *S. mutans* test strains were fluorescently labelled using SYTO9^®^. IgA1 labeled with an Alexa Fluor^®^ Antibody Labeling Kits. Original magnification: ×630. Scale bars, 5 μm (all panels).

Observations by fluorescence microscopy reveal that labeled IgA prominently localized around the SN74 and SN74CNDcomp strains, but localized to a much lesser extent around the MT8148 and CND strains (**Figure 5**).

### Deposition sites of IgA and Cnm in glomeruli, as determined by fluorescent immunostaining

**Figure 6:**
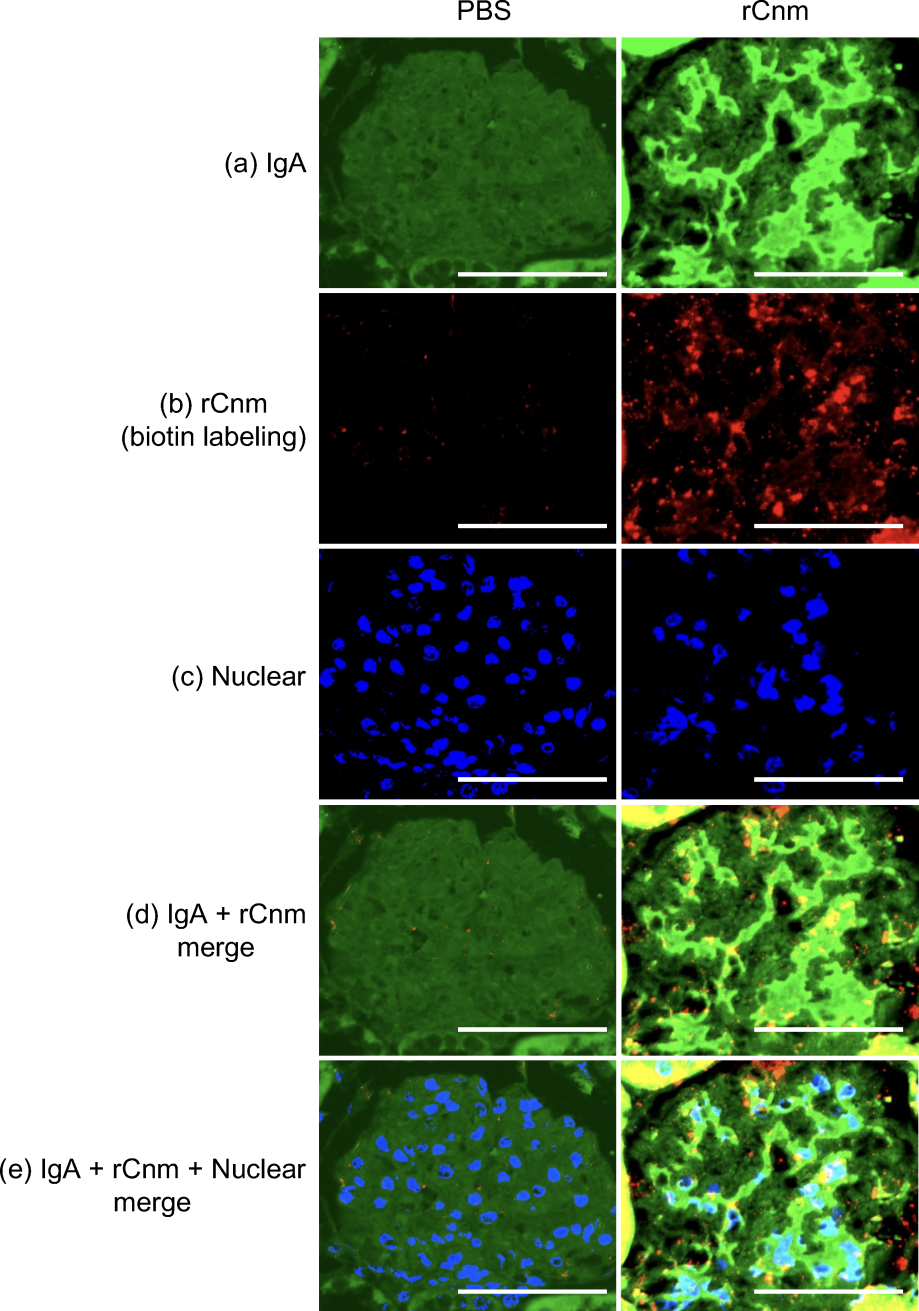
Representative histopathological appearance of kidney tissues by immunohistochemistry with IgA and rCnm (biotin labeling). **(a)** The image shows staining with an anti-IgA antibody. **(b)** The image shows biotin staining. **(c)** The image shows nuclear staining. **(d)** The image shows **(a, b)** images superimposed. **(e)** The image shows **(a–c)** images superimposed. Fluorescein-purified mouse anti-rat IgA (BD Biosciences, Franklin Lakes, NJ, USA) and donkey anti-mouse IgG H&L (Alexa Fluor 488) preadsorbed (ab150109; Abcam) was used for immunofluorescence staining. Original magnification: ×400. Scale bars, 100 μm (all panels).

Immunohistochemical analyses using IgA- and Cnm-specific antibodies showed prominent positive reactions in the mesangial regions of rats injected with rCnm protein. The deposition sites of IgA and Cnm were co-localized. By contrast, these deposits were not observed in rats injected with PBS (**Figure 6**).

## Discussion

Recently, it has become clear that abnormal glycosylation of IgA1 is closely related to the development of IgAN (Suzuki et al., 2008; Gharavi et al., 2008; Novak et al., 2011). There are two subclasses of IgA in human, IgA1 and IgA2, and it has been reported that IgA1 is selectively deposited in the glomeruli of patients with IgAN (Suzuki et al., 2009). Glycosylated IgA1 (galactose-deficient IgA1: Gd-IgA1), in which the galactose of the hinge O-type sugar chain of IgA1 is deleted and N-acetylgalactosamine (GalNAc) is exposed, is increased in the blood of patients with IgAN and has been reported to be a major cause of IgAN (Moldoveanu et al., 2007). However, studies have reported that some patients with IgAN do not develop IgAN despite high blood Gd-IgA1 levels (Gharavi et al., 2008) and that Gd-IgA1 levels do not correlate with urinary protein levels (Moldoveanu et al., 2007). Therefore, Gd-IgA1 alone cannot explain the pathogenesis of IgAN.

Suzuki et al., 2007 established a spontaneous mouse model, Grouped ddY: gddY, which strongly resembles human IgAN, and reported abnormal glycosylation of IgA molecules in these mice. Furthermore, studies have shown that disease activity in murine IgAN correlates with the concentration of IgA immune complexes in the blood (Suzuki et al., 2005, 2007). Blood IgA immune complexes are also increased in patients with human IgAN, and abnormal glycosylation of IgA1, which forms these immune complexes, has been reported (Tomana et al., 1999). It was also found that IgA1-IgG immune complexes extracted from patient serum activated cultured mesangial cells to produce cytokines such as TNF-α and IL-6, which promoted mesangial cell proliferation (Novak et al., 2005). It has been suggested that Gd-IgA1 and its immune complexes may be important in the development and progression of IgAN (Suzuki et al., 2011a; Lai et al., 2009; Camilla et al., 2011; Zhao et al., 2012; Berthoux et al., 2012; Suzuki et al., 2014). It remains unclear where Gd-IgA1 and its corresponding autoantibodies to it are produced and how they form immune complexes.

We have previously revealed that Cnm-positive *S. mutans* is frequently detected in the oral cavity of patients with IgAN (Misaki et al., 2015). We subsequently constructed two animal models, a rat jugular vein model and a rat caries model, and found that Cnm-positive *S. mutans* invades the bloodstream and causes IgAN-like nephritis (Naka et al., 2020, 2022). Recently, in a rat jugular vein model, Cnm protein itself was administered into the blood and found to be expressed in the dens deposit region of the glomerulus (Naka et al., 2024). Our findings suggest that Cnm protein itself may be associated with the development of IgAN, and we hypothesize that Cnm that enters the bloodstream forms a complex with IgA and other immunoglobulins in the blood, adheres to glomeruli, and causes the condition.

The primary objective of this study was to determine the specificity of binding of Cnm to immunoglobulins in various environments after Cnm has entered the bloodstream. Then, the possibility that Cnm forms immune complexes, resulting in glomerular deposition and pathogenesis, was investigated.

*S. mutans* is a gram-positive facultative anaerobe, which is able to grow in both anaerobic and aerobic environments (Hamada and Slade, 1980). This organisms can survive in anaerobic environments by residing within dental plaque and biofilms, whereas in aerobic environments it survives by obtaining energy through fermentation (Yamada, 1987). It has been reported that there is a difference in the growth rate of *S. mutans* when cultured under anaerobic and aerobic conditions (Carlsson et al., 1985). However, there have been no reports comparing the differential expression of *S. mutans* bacterial surface proteins under anaerobic and aerobic conditions. In the blood, oxygen bound to hemoglobin is present, and it is assumed that *S. mutans* that has entered the bloodstream is actively growing in this aerobic environment. The results of qPCR analysis showed that the *cnm* gene expression level of the aerobically-cultured SN74 strain was significantly higher than that of the anaerobically-cultured SN74 strain (**Figure 1A**). This result suggests that the expression level of *cnm* entering the bloodstream may be higher than in the oral cavity or in dental plaque.

Within a biofilm, *S. mutans* metabolizes sucrose to produce acid, which lowers the pH in the biofilm and demineralizes the tooth surface (Matsumoto-Nakano et al., 2007). It is important for the bacteria themselves to be acid-tolerant to continue to grow and produce acid in this low pH environment. *S. mutans* has a higher acid resistance performance than other oral *Streptococcus* species (Takahashi and Yamada, 1999). However, it remained to be determined how different pH environments affect the expression of *S. mutans* bacterial surface proteins.

The pH of the blood is between 7.35 and 7.45, ranging from neutral to moderately alkaline. The expression levels of the *cnm* gene and the Cnm protein in SN74 strains under different pH environments were analyzed using qPCR and western blotting. The results showed that the expression levels of *cnm* and Cnm were higher in environments between pH 7.0 and 7.5 than under acidic or alkaline conditions. Furthermore, observation of the structure of the surface layer of the bacteria of strain SN74 cultured in SEM at between pH 7.0 and 7.5 revealed a marked increase in the number and size of the bumpy structures. These results suggest that the expression of Cnm in cells of strain SN74 that invade the bloodstream may be increased compared with those present in the oral cavity or in dental plaque.

The Cnm protein consists of a collagen-binding domain, a B repeat region, and an LPXTG sequence, and is responsible for binding to the bacterial cell wall (Sato et al., 2004). Our recent report using SEM and immuno-TEM revealed that Cnm proteins are not secreted like other surface proteins, but are bacterially-bound and present on the surface in a projecting form (Naka et al., 2022). However, to date, no studies have examined the possibility of secretion and release from the bacteria under a variety of culture conditions and stress environments.

During incubation, the expression of Cnm in the bacteria decreased, whereas the expression of Cnm in the supernatant of the bacteria tended to increase. These results suggest that Cnm may be secreted or released from the surface layer of the bacteria as the bacteria cells die with prolonged incubation. Correspondingly, *in vivo*, the Cnm of strain SN74 that has invaded the blood may be secreted or released from the bacterial surface over time.

Cnm has been reported to bind to collagen tissue, fibronectin, and fibrinogen in plasma (Otsugu et al., 2017). However, there are no reports on the binding of Cnm to immunoglobulins. ELISA studies showed significantly higher binding of IgA1 to Cnm than to other bacterial surface proteins, and significantly higher bindings of Cnm to IgA1 than to other immunoglobulins. Cnm and IgA1 may have a specific binding relationship, suggesting that Cnm from blood-infected cells of SN74 strain may bind to IgA1 in the blood and form part of an immune complex. Fluorescent imaging of the binding of IgA1 to cultured *S. mutans* revealed that the Cnm-negative MT8148 and CND strains showed only slight binding to IgA1, whereas the Cnm-positive SN74 and Comp strains showed significant binding between the bacteria and IgA1. It was suggested that Cnm expressed on the surface layer of the bacteria may be involved in binding to IgA1. Furthermore, in the glomeruli of rCnm-treated rats, there was significant IgA deposition and Cnm deposition in the mesangial region. These results suggest that IgA and Cnm may bind, form immune complexes, and deposit in glomeruli.

In summary, the results of the present study suggest that after Cnm enters the bloodstream, it binds to IgA1 and forms immune complexes, which are deposited in the glomerular mesangial region and may contribute to the development of IgAN. In future studies, we will continue to elucidate the role of Cnm in IgAN development.

## Data Availability

The datasets presented in this study can be found in online repositories. The names of the repository/repositories and accession number(s) can be found in the article/**Supplementary Material**.

## References

[B1] Abranches J. Miller J. H. Martinez A. R. Simpson-Haidaris P. J. Burne R. A. Lemos J. A. (2011). The collagen-binding protein Cnm is required for *Streptococcus mutans* adherence to and intracellular invasion of human coronary artery endothelial cells. Infect. Immun. 79, 2277–2284. doi: 10.1128/IAI.00767-10, PMID: 21422186 PMC3125845

[B2] Berger J. (1969). IgA glomerular deposits in renal disease. Transplant. Proc. 1, 939–944. 4107073

[B3] Berthoux F. Suzuki H. Thibaudin L. Yanagawa H. Maillard N. Mariat C. . (2012). Autoantibodies targeting galactose-deficient IgA1 associate with progression of IgA nephropathy. J. Am. Soc. Nephrol. 23, 1579–1587. doi: 10.1681/ASN.2012010053, PMID: 22904352 PMC3431415

[B4] Camilla R. Suzuki H. Dapra V. Loiacono E. Peruzzi L. Amore A. . (2011). Oxidative stress and galactose-deficient IgA1 as markers of progression in IgA nephropathy. Clin. J. Am. Soc. Nephrol. 6, 1903–1911. doi: 10.2215/CJN.11571210, PMID: 21784819 PMC3156425

[B5] Carlsson J. Kujala U. Edlund M. B. (1985). Pyruvate dehydrogenase activity in *Streptococcus mutans*. Infect. Immun. 49, 674–678. doi: 10.1128/iai.49.3.674-678.1985, PMID: 4030096 PMC261240

[B6] Coppo R. Amore A. Peruzzi L. Vergano L. Camilla R. (2010). Innate immunity and IgA nephropathy. J. Nephrol. 23, 626–632. 20383870

[B7] Di Cologna N. M. Samaddar S. Valle C. A. Vargas J. Aviles-Reyes A. Morales J. . (2021). Amyloid aggregation of *Streptococcus mutans cnm* influences its collagen-binding activity. Appl. Environ. Microbiol. 87, e0114921. doi: 10.1128/AEM.01149-21, PMID: 34406827 PMC8516039

[B8] Donadio J. V. Grande J. P. (2002). IgA nephropathy. N Engl. J. Med. 347, 738–748. doi: 10.1056/NEJMra020109, PMID: 12213946

[B9] Fujiwara T. Nakano K. Kawaguchi M. Ooshima T. Sobue S. Kawabata S. . (2001). Biochemical and genetic characterization of serologically untypable *Streptococcus mutans* strains isolated from patients with bacteremia. Eur. J. Oral. Sci. 109, 330–334. doi: 10.1034/j.1600-0722.2001.00119.x, PMID: 11695754

[B10] Gharavi A. G. Moldoveanu Z. Wyatt R. J. Barker C. V. Woodford S. Y. Lifton R. P. . (2008). Aberrant IgA1 glycosylation is inherited in familial and sporadic IgA nephropathy. J. Am. Soc. Nephrol. 19, 1008–1014. doi: 10.1681/ASN.2007091052, PMID: 18272841 PMC2386728

[B11] Gregory M. C. Hammond M. E. Brewer E. D. (1988). Renal deposition of cytomegalovirus antigen in immunoglobulin-A nephropathy. Lancet 1, 11–14. doi: 10.1016/S0140-6736(88)91000-8, PMID: 2891887

[B12] Hamada S. Slade H. D. (1980). Biology, immunology, and cariogenicity of *Streptococcus mutans*. Microbiol. Rev. 44, 331–384. doi: 10.1128/mr.44.2.331-384.1980, PMID: 6446023 PMC373181

[B13] Iwama H. Horikoshi S. Shirato I. Tomino Y. (1998). Epstein-Barr virus detection in kidney biopsy specimens correlates with glomerular mesangial injury. Am. J. Kidney Dis. 32, 785–793. doi: 10.1016/S0272-6386(98)70134-9, PMID: 9820448

[B14] Koyama A. Sharmin S. Sakurai H. Shimizu Y. Hirayama K. Usui J. . (2004). Staphylococcus aureus cell envelope antigen is a new candidate for the induction of IgA nephropathy. Kidney Int. 66, 121–132. doi: 10.1111/j.1523-1755.2004.00714.x, PMID: 15200419

[B15] Lai K. N. Leung J. C. Chan L. Y. Saleem M. A. Mathieson P. W. Tam K. Y. . (2009). Podocyte injury induced by mesangial-derived cytokines in IgA nephropathy. Nephrol. Dial Transplant. 24, 62–72. doi: 10.1093/ndt/gfn441, PMID: 18685143

[B16] Matsumi Y. Fujita K. Takashima Y. Yanagida K. Morikawa Y. Matsumoto-Nakano M. (2015). Contribution of glucan-binding protein A to firm and stable biofilm formation by *Streptococcus mutans*. Mol. Oral. Microbiol. 30, 217–226. doi: 10.1111/omi.12085, PMID: 25256943

[B17] Matsumoto-Nakano M. Kuramitsu H. K. (2006). Role of bacteriocin immunity proteins in the antimicrobial sensitivity of *Streptococcus mutans*. J. Bacteriol. 188, 8095–8102. doi: doi: 10.1128/JB.00908-06, PMID: 16997961 PMC1698205

[B18] Matsumoto-Nakano M. Fujita K. Ooshima T. (2007). Comparison of glucan-binding proteins in cariogenicity of *Streptococcus mutans*. Oral. Microbiol. Immunol. 22, 30–35. doi: 10.1111/j.1399-302X.2007.00318.x, PMID: 17241168

[B19] Matsumoto-Nakano M. Tsuji M. Amano A. Ooshima T. (2008). Molecular interactions of alanine-rich and proline-rich regions of cell surface protein antigen c in *Streptococcus mutans*. Oral. Microbiol. Immunol. 23, 265–270. doi: 10.1111/j.1399-302X.2007.00421.x, PMID: 18582324

[B20] Misaki T. Naka S. Kuroda K. Nomura R. Shiooka T. Naito Y. . (2015). Distribution of *Streptococcus mutans* strains with collagen-binding proteins in the oral cavity of IgA nephropathy patients. Clin. Exp. Nephrol. 19, 844–850. doi: 10.1007/s10157-014-1072-0, PMID: 25492252

[B21] Moldoveanu Z. Wyatt R. J. Lee J. Y. Tomana M. Julian B. A. Mestecky J. . (2007). Patients with IgA nephropathy have increased serum galactose-deficient IgA1 levels. Kidney Int. 71, 1148–1154. doi: 10.1038/sj.ki.5002185, PMID: 17342176

[B22] Naka S. Hatakeyama R. Takashima Y. Matsumoto-Nakano M. Nomura R. Nakano K. (2016). Contributions of *Streptococcus mutans* Cnm and PA antigens to aggravation of non-alcoholic steatohepatitis in mice. Sci. Rep. 6, 36886. doi: 10.1038/srep36886, PMID: 27833139 PMC5105074

[B23] Naka S. Matsuoka D. Goto K. Misaki T. Nagasawa Y. Ito S. . (2022). Cnm of *Streptococcus mutans* is important for cell surface structure and membrane permeability. Front. Cell Infect. Microbiol. 12, 994014. doi: 10.3389/fcimb.2022.994014, PMID: 36176579 PMC9513430

[B24] Naka S. Matsuoka D. Misaki T. Nagasawa Y. Ito S. Nomura R. . (2024). Contribution of collagen-binding protein Cnm of *Streptococcus mutans* to induced IgA nephropathy-like nephritis in rats. Commun. Biol. 7, 1141. doi: 10.1038/s42003-024-06826-x, PMID: 39277690 PMC11401903

[B25] Naka S. Nomura R. Takashima Y. Okawa R. Ooshima T. Nakano K. (2014). A specific *Streptococcus mutans* strain aggravates non-alcoholic fatty liver disease. Oral. Dis. 20, 700–706. doi: 10.1111/odi.12191, PMID: 25360469

[B26] Naka S. Wato K. Hatakeyama R. Okawa R. Nomura R. Nakano K. (2018). Longitudinal comparison of *Streptococcus mutans*-induced aggravation of non-alcoholic steatohepatitis in mice. J. Oral. Microbiol. 10, 1428005. doi: 10.1080/20002297.2018.1428005, PMID: 29503703 PMC5795759

[B27] Naka S. Wato K. Misaki T. Ito S. Matsuoka D. Nagasawa Y. . (2021). *Streptococcus mutans* induces IgA nephropathy-like glomerulonephritis in rats with severe dental caries. Sci. Rep. 11, 5784. doi: 10.1038/s41598-021-85196-4, PMID: 33707585 PMC7952735

[B28] Naka S. Wato K. Misaki T. Ito S. Nagasawa Y. Nomura R. . (2020). Intravenous administration of *Streptococcus mutans* induces IgA nephropathy-like lesions. Clin. Exp. Nephrol. 24, 1122–1131. doi: 10.1007/s10157-020-01961-1, PMID: 32909181 PMC7599197

[B29] Nakano K. Hokamura K. Taniguchi N. Wada K. Kudo C. Nomura R. . (2011). The collagen-binding protein of *Streptococcus mutans* is involved in haemorrhagic stroke. Nat. Commun. 2, 485. doi: 10.1038/ncomms1491, PMID: 21952219 PMC3220351

[B30] Nakano K. Lapirattanakul J. Nomura R. Nemoto H. Alaluusua S. Gronroos L. . (2007). *Streptococcus mutans* clonal variation revealed by multilocus sequence typing. J. Clin. Microbiol. 45, 2616–2625. doi: 10.1128/JCM.02343-06, PMID: 17567784 PMC1951271

[B31] Nakano K. Nomura R. Nakagawa I. Hamada S. Ooshima T. (2004). Demonstration of *Streptococcus mutans* with a cell wall polysaccharide specific to a new serotype, k, in the human oral cavity. J. Clin. Microbiol. 42, 198–202. doi: 10.1128/JCM.42.1.198-202.2004, PMID: 14715753 PMC321689

[B32] Nakano K. Ooshima T. (2009). Serotype classification of *Streptococcus mutans* and its detection outside the oral cavity. Future Microbiol. 4, 891–902. doi: 10.2217/fmb.09.64, PMID: 19722842

[B33] Nomura R. Naka S. Nemoto H. Inagaki S. Taniguchi K. Ooshima T. . (2013). Potential involvement of collagen-binding proteins of *Streptococcus mutans* in infective endocarditis. Oral. Dis. 19, 387–393. doi: 10.1111/odi.12016, PMID: 22998492

[B34] Nomura R. Nakano K. Ooshima T. (2005). Molecular analysis of the genes involved in the biosynthesis of serotype specific polysaccharide in the novel serotype k strains of *Streptococcus mutans*. Oral. Microbiol. Immunol. 20, 303–309. doi: 10.1111/j.1399-302X.2005.00231.x, PMID: 16101966

[B35] Nomura R. Otsugu M. Hamada M. Matayoshi S. Teramoto N. Iwashita N. . (2020). Potential involvement of *Streptococcus mutans* possessing collagen binding protein Cnm in infective endocarditis. Sci. Rep. 10, 19118. doi: 10.1038/s41598-020-75933-6, PMID: 33154489 PMC7645802

[B36] Novak J. Moldoveanu Z. Julian B. A. Raska M. Wyatt R. J. Suzuki Y. . (2011). Aberrant glycosylation of IgA1 and anti-glycan antibodies in IgA nephropathy: role of mucosal immune system. Adv. Otorhinolaryngol 72, 60–63. doi: 10.1159/000324607, PMID: 21865691

[B37] Novak J. Tomana M. Matousovic K. Brown R. Hall S. Novak L. . (2005). IgA1-containing immune complexes in IgA nephropathy differentially affect proliferation of mesangial cells. Kidney Int. 67, 504–513. doi: 10.1111/j.1523-1755.2005.67107.x, PMID: 15673298

[B38] Ooshima T. Izumitani A. Sobue S. Okahashi N. Hamada S. (1983). Non-cariogenicity of the disaccharide palatinose in experimental dental caries of rats. Infect. Immun. 39, 43–49. doi: 10.1128/iai.39.1.43-49.1983, PMID: 6822422 PMC347905

[B39] Otsugu M. Nomura R. Matayoshi S. Teramoto N. Nakano K. (2017). Contribution of *Streptococcus mutans* strains with collagen-binding proteins in the presence of serum to the pathogenesis of infective endocarditis. Infect. Immun. 85, 12. doi: 10.1128/IAI.00401-17, PMID: 28947650 PMC5695098

[B40] Sato Y. Okamoto K. Kagami A. Yamamoto Y. Igarashi T. Kizaki H. (2004). *Streptococcus mutans* strains harboring collagen-binding adhesin. J. Dent. Res. 83, 534–539. doi: 10.1177/154405910408300705, PMID: 15218042

[B41] Schmitt R. Lindahl G. Karpman D. (2010). Antibody response to IgA-binding streptococcal M proteins in children with IgA nephropathy. Nephrol. Dial Transplant. 25, 3434–3436. doi: 10.1093/ndt/gfq346, PMID: 20558662

[B42] Suzuki H. Fan R. Zhang Z. Brown R. Hall S. Julian B. A. . (2009). Aberrantly glycosylated IgA1 in IgA nephropathy patients is recognized by IgG antibodies with restricted heterogeneity. J. Clin. Invest. 119, 1668–1677. doi: 10.1172/JCI38468, PMID: 19478457 PMC2689118

[B43] Suzuki H. Kiryluk K. Novak J. Moldoveanu Z. Herr A. B. Renfrow M. B. . (2011a). The pathophysiology of IgA nephropathy. J. Am. Soc. Nephrol. 22, 1795–1803. doi: 10.1681/ASN.2011050464, PMID: 21949093 PMC3892742

[B44] Suzuki Y. Matsuzaki K. Suzuki H. Okazaki K. Yanagawa H. Ieiri N. . (2014). Serum levels of galactose-deficient immunoglobulin (Ig) A1 and related immune complex are associated with disease activity of IgA nephropathy. Clin. Exp. Nephrol. 18, 770–777. doi: 10.1007/s10157-013-0921-6, PMID: 24477513 PMC4194014

[B45] Suzuki H. Moldoveanu Z. Hall S. Brown R. Vu H. L. Novak L. . (2008). IgA1-secreting cell lines from patients with IgA nephropathy produce aberrantly glycosylated IgA1. J. Clin. Invest. 118, 629–639. doi: 10.1172/JCI33189, PMID: 18172551 PMC2157566

[B46] Suzuki S. Nakatomi Y. Sato H. Tsukada H. Arakawa M. (1994). Haemophilus parainfluenzae antigen and antibody in renal biopsy samples and serum of patients with IgA nephropathy. Lancet 343, 12–16. doi: 10.1016/S0140-6736(94)90875-3, PMID: 7905040

[B47] Suzuki H. Suzuki Y. Aizawa M. Yamanaka T. Kihara M. Pang H. . (2007). Th1 polarization in murine IgA nephropathy directed by bone marrow-derived cells. Kidney Int. 72, 319–327. doi: 10.1038/sj.ki.5002300, PMID: 17495863

[B48] Suzuki Y. Suzuki H. Nakata J. Sato D. Kajiyama T. Watanabe T. . (2011b). Pathological role of tonsillar B cells in IgA nephropathy. Clin. Dev. Immunol. 2011, 639074. doi: 10.1155/2011/639074, PMID: 21785618 PMC3139900

[B49] Suzuki H. Suzuki Y. Yamanaka T. Hirose S. Nishimura H. Toei J. . (2005). Genome-wide scan in a novel IgA nephropathy model identifies a susceptibility locus on murine chromosome 10, in a region syntenic to human IGAN1 on chromosome 6q22-23. J. Am. Soc. Nephrol. 16, 1289–1299. doi: 10.1681/ASN.2004030219, PMID: 15772254

[B50] Takahashi N. Yamada T. (1999). Acid-induced acid tolerance and acidogenicity of non-mutans streptococci. Oral. Microbiol. Immunol. 14, 43–48. doi: 10.1034/j.1399-302X.1999.140105.x, PMID: 10204479

[B51] Takashima Y. Fujita K. Ardin A. C. Nagayama K. Nomura R. Nakano K. . (2015). Characterization of the dextran-binding domain in the glucan-binding protein C of *Streptococcus mutans*. J. Appl. Microbiol. 119, 1148–1157. doi: 10.1111/jam.12895, PMID: 26176557

[B52] Tomana M. Novak J. Julian B. A. Matousovic K. Konecny K. Mestecky J. (1999). Circulating immune complexes in IgA nephropathy consist of IgA1 with galactose-deficient hinge region and antiglycan antibodies. J. Clin. Invest. 104, 73–81. doi: 10.1172/JCI5535, PMID: 10393701 PMC408399

[B53] Waldo F. B. Britt W. J. Tomana M. Julian B. A. Mestecky J. (1989). Non-specific mesangial staining with antibodies against cytomegalovirus in immunoglobulin-A nephropathy. Lancet 1, 129–131. doi: 10.1016/S0140-6736(89)91144-6, PMID: 2463443

[B54] Wyatt R. J. Julian B. A. (2013). IgA nephropathy. N Engl. J. Med. 368, 2402–2414. doi: 10.1056/NEJMra1206793, PMID: 23782179

[B55] Yamada T. (1987). The role of ribosomes in the sensitivity of mycobacteria to tuberactinomycin. Microbiol. Immunol. 31, 179–181. doi: 10.1111/j.1348-0421.1987.tb03081.x, PMID: 3037284

[B56] Yeo S. C. Cheung C. K. Barratt J. (2018). New insights into the pathogenesis of IgA nephropathy. Pediatr. Nephrol. 33, 763–777. doi: 10.1007/s00467-017-3699-z, PMID: 28624979 PMC5861174

[B57] Yuan K. Hou L. Jin Q. Niu C. Mao M. Wang R. . (2021). Comparative transcriptomics analysis of *Streptococcus mutans* with disruption of LuxS/AI-2 quorum sensing and recovery of methyl cycle. Arch. Oral. Biol. 127, 105137. doi: 10.1016/j.archoralbio.2021.105137, PMID: 33965851

[B58] Zhao N. Hou P. Lv J. Moldoveanu Z. Li Y. Kiryluk K. . (2012). The level of galactose-deficient IgA1 in the sera of patients with IgA nephropathy is associated with disease progression. Kidney Int. 82, 790–796. doi: 10.1038/ki.2012.197, PMID: 22673888 PMC3443545

