# Supplementary figures and images for: Binding of IgA1 and surface-expressed collagen-binding protein of Streptococcus mutans contributes to IgA nephropathy pathogenesis
Source: Front Cell Infect Microbiol. 2026 Jan 7;15:1673581. doi: 10.3389/fcimb.2025.1673581 (PMC12819641; doi:10.3389/fcimb.2025.1673581)

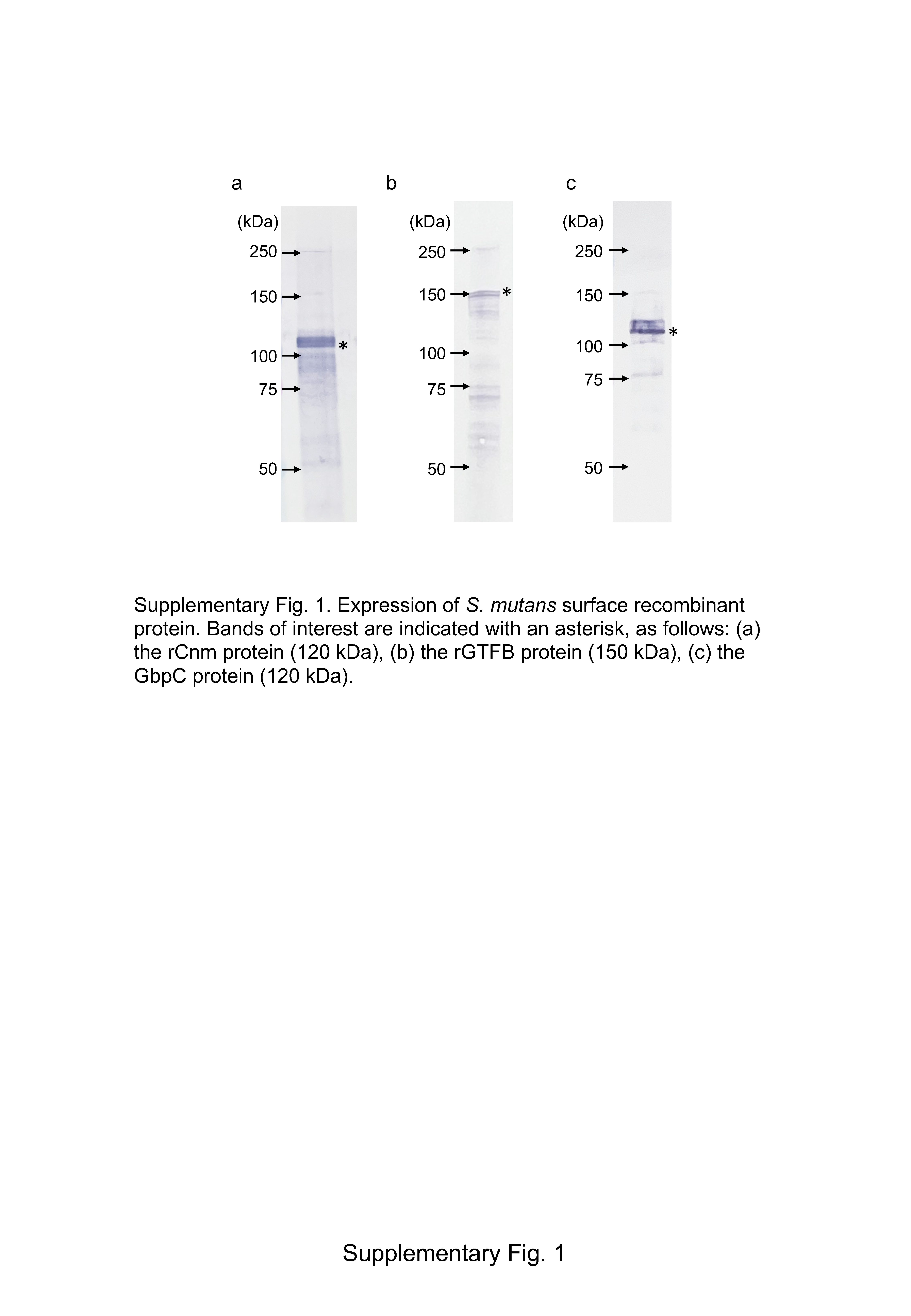

Supplement: Supplementary file 1 [file Image1.tiff]

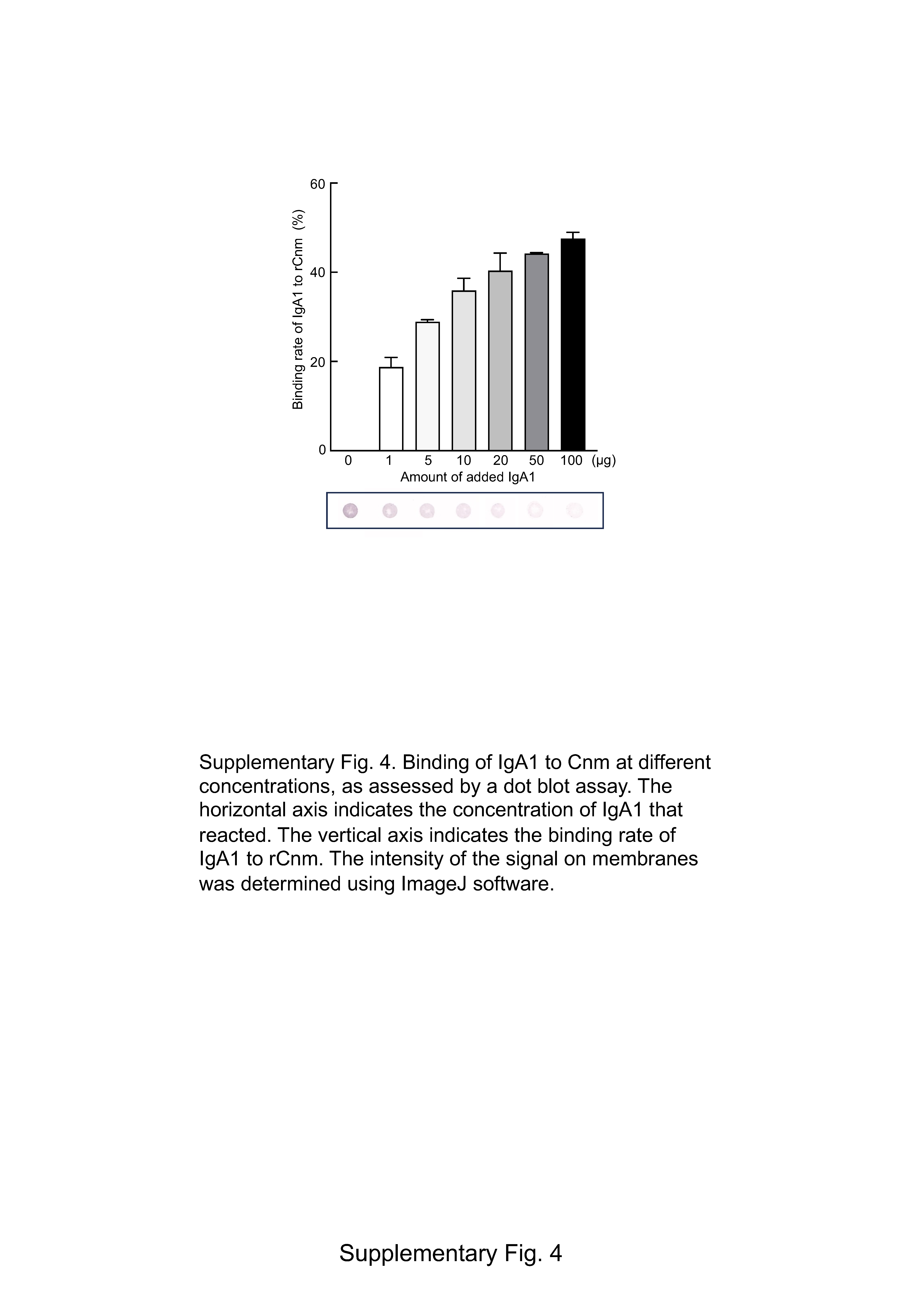

Supplement: Supplementary file 4 [file Image4.tiff]
